# Endogenous FGF1 Deficiency Aggravates Doxorubicin-Induced Hepatotoxicity

**DOI:** 10.3390/toxics11110925

**Published:** 2023-11-12

**Authors:** Chunjie Gu, Zijuan Liu, Yingjian Li, Mei Yi, Simeng Wang, Xia Fan, Da Sun, Chi Zhang, Xiaoqing Yan, Guicheng Wu

**Affiliations:** 1 The Chinese-American Research Institute for Diabetic Complications, School of Pharmaceutical Sciences, Wenzhou Medical University, Wenzhou 325035, China; 2 Department of Clinical Translational Research, The Third Affiliated Hospital of Wenzhou Medical University, Wenzhou 325200, China; 3 Institute of Life Sciences, Wenzhou University, Wenzhou 325200, China; 4 Department of Hepatology, Chongqing University Three Gorges Hospital, Chongqing 404000, China; 5 Chongqing Municipality Clinical Research Center for Endocrine and Metabolic Diseases, Chongqing 400015, China; 6 School of Medicine, Chongqing University, Chongqing 400030, China

**Keywords:** doxorubicin, hepatoxicity, FGF-1, oxidative stress, apoptosis, fibrosis

## Abstract

Doxorubicin (DOX) is a broad-spectrum antineoplastic agent that widely used in clinic. However, its application is largely limited by its toxicity in multiple organs. Fibroblast growth factor 1 (FGF1) showed protective potential in various liver diseases, but the role of endogenous FGF1 in DOX-induced liver damage is currently unknown. Both wild-type (WT) and FGF1 knockout (FGF1-KO) mice were treated with DOX. DOX induced loss of body weight and liver weight and elevation of ALT and AST in WT mice, which were aggravated by FGF1 deletion. FGF1 deletion exacerbated hepatic oxidative stress mirrored by further elevated 3-nitrosative modification of multiple proteins and malondialdehyde content. These were accompanied by blunted compensatively antioxidative responses indicated by impaired upregulation of nuclear factor erythroid 2-related factor 2 and its downstream antioxidant gene expression. The aggravated oxidative stress was coincided with exacerbated cell apoptosis in DOX-treated FGF1-KO mice reflected by further increased TUNEL positive cell staining and BCL-2-associated X expression and caspase 3 cleavage. These detrimental changes in DOX-treated FGF1-KO mice were associated with worsened intestinal fibrosis and increased upregulation fibrotic marker connective tissue growth factor and α-smooth muscle actin expression. However, DOX-induced hepatic inflammatory responses were not further affected by FGF1 deletion. These results demonstrate that endogenous FGF1 deficiency aggravates DOX-induced liver damage and FGF1 is a potential therapeutic target for treatment of DOX-associated hepatoxicity.

## 1. Introduction

Doxorubicin (DOX, also known as Adriamycin) is an anthracycline antibiotic that is widely used as an effective chemotherapeutic drug to treat several forms of cancer, including acute leukemia, lymphoma, sarcoma and solid tumors [1,2]. However, its clinical use is largely limited by its adverse effects [3,4]. The liver is the major organ involved in the process of metabolism and detoxification of DOX, which makes the liver suffered the toxic effects of DOX [5]. The hepatotoxicity of DOX has been observed in clinical patients [6], and alleviating the hepatotoxicity of DOX becomes a practical issue during its clinical applications. One potential strategy is to improve the formulation of DOX. Liposome-encapsulated form of doxorubicin showed to be less toxic than free doxorubicin. Another promising strategy is to develop agents that can directly prevent DOX-induced hepatotoxicity, which needs a better understanding of the pathogenesis involved. The molecular mechanisms underlying DOX-induced hepatotoxicity are thought to be due to oxidative stress resulted from the imbalance between the overproduction of DOX-induced reactive oxygen species (ROS) and insufficiency of endogenous redox potential, which occurs during DOX metabolism in the liver and leads to lipid peroxidation, mitochondrial dysfunction, and inflammation [5]. These eventually cause hepatocyte death and leakage of hepatic enzymes into the circulation [7]. Various types of natural products and certain drugs have shown to produce a hepatoprotective effect eliminating the toxic effect of doxorubicin [5]. However, there are no treatment strategies available that specifically target DOX-induced hepatotoxicity in large part because the pathology of DOX-induced hepatotoxicity is inadequately understood.

Fibroblast growth factor 1 (FGF1) is a well characterized and multifunctional mitogen belonging to the FGF superfamily. FGF1 is expressed in multiple organs, including brain, liver, kidney, and heart [8,9,10,11,12,13,14,15], and plays a number of roles in development, regeneration, and angiogenesis. It is widely studied for its therapeutic benefits in cardiovascular disorders, nerve injury and wound healing [16,17]. However, the pathogenic roles of endogenous FGF1 in DOX-induced hepatotoxicity remain untested.

In the present study, the potential role of FGF1 in DOX-induced liver damage was investigated in FGF1 knockout (FGF1-KO) mice with chronic DOX administration. 

## 2. Materials and Methods

### 2.1. Animals and Treatment

FGF1-KO mice with C57BL/6J background were established as described in previous report [18], and the littermate wide type (WT) mice were used as controls. All mice were housed under a 12 h light and dark cycle and 22 ± 2 °C conditions. Nine-week-old male WT and FGF1-KO mice were both randomly assigned to two groups to receive intraperitoneal injection of either DOX (5 mg/kg body weight, MedChemExpress, Monmouth Junction, NJ, USA) or phosphate-buffered saline (PBS) once a week for consecutive five weeks as described previously [19], i.e., WT PBS (*n* = 6), WT DOX (*n* = 10), FGF1-KO PBS (*n* = 5), and FGF1-KO DOX (*n* = 11). DOX was dissolved in PBS at a final concentration of 0.5 mg/mL and an appropriate volume was injected proportionally to body weight. The DOX dose of 5 mg/kg (15 mg/m^2^) for consecutive 5 weeks equals an accumulative dose of 25 mg/kg (75 mg/m^2^) that is equivalent to a dose what is typically given to breast cancer patients [20]. One week after the last injection, mice were euthanized and the left lateral lobe of each liver was harvested and cut into two pieces along the short axis for biochemical and histological assays, respectively. Rodent feed and tap water were supplied by Experiment Animal Center of Wenzhou Medical University. All experimental procedures were in accordance with Laboratory Animal Guide and Policy of Wenzhou Medical University and approved by the Institutional Animal Care and Use Committee of the Wenzhou Medical University.

### 2.2. Alanine Aminotransferase (ALT) and Aspartate Transaminase (AST) Assay

The plasma levels of ALT and AST were measured using commercially available kits (Cayman Chemical, Ann Arbor, MI, USA) according to the manufacturer’s instructions. Briefly, 150 μL of ALT or AST substrate, 20 μL of ALT or AST cofactor, and 20 μL sample were added into the wells of a 96-well plate. After incubation at 37 °C for 15 min, 20 μL of ALT or AST Initiator was added and the absorbance at 340 nm was measured once every minute for 10 min at 37 °C.

### 2.3. RNA Extraction, cDNA Synthesis and Quantitative Real-Time Reverse Transcription PCR (qRT-PCR)

Total RNA was extracted from liver tissue using TRIzol^TM^ reagent (Invitrogen, Carlsbad, CA, USA), and reverse-transcribed to cDNA using reverse transcription kit (Promega, Madison, WI, USA) following the manufacturer’s instructions. qRT-PCR reactions were performed on a Roche LightCycler 96 (Roche, Basel, Switzerland). Glyceraldehyde 3-phosphate dehydrogenase (GAPDH) was used as an internal loading control. All TaqMan primers were purchased from Thermo Fisher Scientific Inc. (Grand Island, NY, USA). Catalog numbers of the primers were listed as follows: GAPDH, Mm99999915_g1; transforming growth factor β (TGFβ), Mm01178820_m1; connective tissue growth factor (CTGF), Mm01192933_g1; α-smooth muscle actin (α-SMA), Mm01546133_m1. Superoxide dismutase type 1 (SOD1), Mm00809556_s1, intercellular adhesion molecule 1 (ICAM1), Mm00516023_m1; vascular cell adhesion molecule 1 (VCAM1), Mm01320970_m1, monocyte chemoattractant protein-1 (MCP-1), Mm00441242_m1, nuclear factor erythroid 2-related factor 2 (NRF2), Mm004777784_m1, NAD(P)H dehydrogenase [quinone] 1 (NQO1), Mm01253561_m1, and heme oxygenase 1 (HO1), Mm00516005_m1.

### 2.4. Liver Histopathological Analysis

Liver tissues were fixed overnight in 10% formalin and embedded in paraffin. After deparaffinization and rehydration, sections were subjected to Sirius Red staining as described previously [21] to observe the collagen accumulation in interstitial and perivascular areas of liver tissues for evaluating hepatic fibrosis. The positively stained areas were quantified by Image J (NIH, Bethesda, MD, USA) and normalized to the WT control mice.

### 2.5. Immunohistochemical Staining

Immunohistochemical staining of CD68 and Ly6G was performed to detect inflammatory cell infiltration. In brief, the deparaffinized and rehydrated liver sections were incubated with 0.3% hydrogen peroxide for 30 min to quench endogenous peroxidase activity, and then washed with ddH_2_O and incubated in Tris-ethylenediaminetetraacetic acid (EDTA) buffer at 95 °C for 15 min for antigen retrieval. After returning to room temperature, sections were then blocked with 5% bovine serum albumin (BSA) for 30 min at room temperature. Thereafter, sections were incubated with rabbit anti-mouse CD68 (1:1000, Abcam, Waltham, MA, USA) or Ly6G [1:1000, Cell Signaling Technology (CST), Danvers, MA, USA] antibody diluted in 5% BSA overnight at 4 °C in a humid chamber. After washing three times with PBS containing 0.1% Tween^®^ 20 (PBST), sections were incubated with horseradish peroxidase-conjugated goat anti-rabbit IgG and visualized using 3,3-diaminobenzidine kit (Vector Laboratories, Inc., Newark, CA, USA, Cat # SK-4100) following the manufacturer’s instructions, and then counterstained with hematoxylin. Inflammatory cell infiltration was expressed as CD68^+^ or Ly6G^+^ cells per 40× field.

### 2.6. Terminal Deoxynucleotidyl Transferase dUTP Nick End Labeling (TUNEL) Assay

Apoptosis was detected by TUNEL staining (Promega, Madison, WI, USA, Cat # G3250) per the manufacturer’s instructions. In brief, the deparaffinized and rehydrated liver sections were fixed in 4% formaldehyde solution for 15 min and washed with PBS and permeabilized with a Proteinase K solution at room temperature for 10 min. After repeated fixation with 4% formaldehyde solution for 5 min and washing with PBS, specimens were equilibrated by covering with equilibration buffer at room temperature for 10 min, followed by addition of TdT reaction mixture for 60 min at 37 °C in a humidified chamber. Then, sections were immersed in 2× saline sodium citrate buffer for 15 min and washed with PBS and counterstained with 4′,6-diamidino-2-245 phenylindole (DAPI) for recognizing nuclei. Images were captured by a fluorescent microscope (Olympus, Japan) with 40× magnification and analyzed by counting TUNEL positive nuclei.

### 2.7. Western Blot Analysis

The liver tissues were homogenized in radioimmunoprecipitation assay buffer (CST) at 4 °C for 4 h, and then centrifuged at 12,000 rpm at 4 °C for 25 min. The supernatant was collected, and the protein concentration was detected using a Protein Assay Dye Reagent Concentrate (Bio-Rad, Hercules, CA, USA). Total proteins (40 μg) were separated by 8–12% sodium dodecyl sulfate (SDS) polyacrylamide-gel electrophoresis using a Miniprotein system (Bio-Rad) and electro-transferred onto 0.22 μm nitrocellulose (NC) membranes (Millipore, Burlington, MA, USA). The NC membranes were blocked with 5% defatted milk for 1 h at room temperature and then incubated overnight with rabbit anti-mouse antibodies against FGF1 (Abcam,1:1000), 3-nitrotyrosine (3-NT, Millipore, 1;1000), ICAM1 (Proteintech, Rosemont, IL, USA, 1:1000), VCAM1 (1:1000, CST), MCP1 (1:1000, CST), Bcl2-associated X (BAX, ABclonal Technology, Woburn, MA, USA, 1:1000), proliferating cell nuclear antigen (PCNA, 1:1000, CST), cleaved-caspase 3 (1:1000, CST), GAPDH (1:1000, CST) or α-tubulin (1:1000, CST) at 4 °C overnight. After three washes with tris-buffered saline with 0.1% Tween^®^ 20 (TBST), NC membranes were incubated with peroxidase-conjugated goat anti-rabbit IgG secondary antibody (1:3000, CST) at room temperature for 1 h. Then, the membranes were washed three times with TBST, and the chromogen was developed in enhanced chemiluminescence reagent (Bio-Rad) and visualized under a ChemiDoc^TM^ MP Imaging System (Bio-Rad). The densities of the antibody recognized protein bands were quantified using ImageJ (NIH) and GAPDH or α-Tubulin as a loading control. Each blot includes an identical sample as a control for quantitative analysis among multiple blots.

### 2.8. Malondiadehyde (MDA) Content Assay

Liver tissues were homogenized in lysis buffer on ice. After centrifugation at 12,000 rpm at 4 °C for 20 min, the supernatant was collected. The supernatant (50 μL) was incubated with 20 μL SDS (8.1%), 150 μL acetic acid (20%) and 210 μL thiobarbituric acid (0.57%) at 90 °C for 70 min. After cooling down in ice bath, 100 μL double-distilled water was added into each sample. Then, the samples were centrifugated at 4000 rpm for 15 min and the supernatants were collected. The optical density (OD) value of each sample was detected by a microplate reader (SpectraMax M3, Molecular Devices, Sunnyvale, CA, USA) at 540 nm. The final MDA content in each sample was calculated based on the equation: [MDA] = (OD of Sample–OD of Blank) *178/protein concentration (mmol/mg). The MDA content was normalized by protein concentration.

### 2.9. Statistical Analysis

Data were presented as the means ± SEM and were analyzed using GraphPad Prism version 9 software. For the multiple groups data, we performed two-way ANOVA followed by Tukey’s multiple comparison test. For the two independent samples, when the data followed a Gaussian distribution, an unpaired, two-tailed Student’s test was used; when the data did not follow a Gaussian distribution, we used the Mann–Whitney U test. Differences were considered statistically significant at *p* < 0.05.

## 3. Results

### 3.1. FGF1 Deficiency Slightly Aggravates DOX-Induced Liver Damage

**Figure 1:**
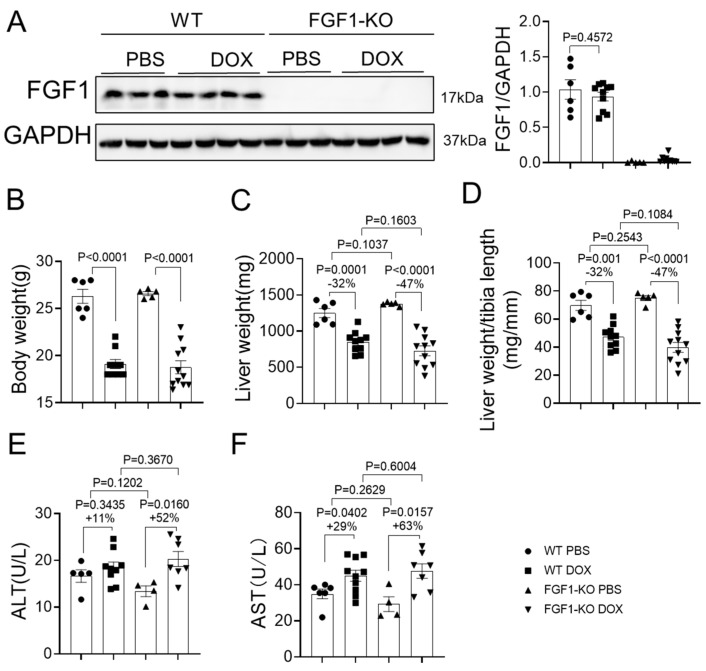
FGF1 deficiency aggravated DOX-induced liver damage. Nine-week-old wild-type (WT) and FGF1-KO mice were treated with DOX (5 mg/kg body weight) or PBS vehicle once a week for consecutive five weeks. (**A**) FGF1 expression was detected by Western blot and quantified by densitometry. (**B**) Body weight. (**C**) Liver weight. (**D**) The ratio of liver weight to tibial length. (**E**,**F**) ALT and AST levels in plasma. Values represent the mean ± SEM. *p* < 0.05 was considered statistically significant. The percentage under the *p* values indicated the magnitude of either decrease (−) or increase (+) between two groups.

The efficiency of FGF1 deletion was validated by Western blot and the results indicated a complete FGF1 deficiency in the liver of FGF1-KO mice; however, DOX treatment had no significant effects on endogenous FGF1 expression in the liver of WT mice (Figure 1A). Compared to PBS treated controls, DOX treatment dramatically decreased body weight in both WT and FGF1-KO mice, while no significant differences were observed between WT and FGF1-KO mice either with or without DOX treatment (Figure 1B). The direct effects of DOX on liver were evaluated by measuring liver weight (Figure 1C) and normalizing liver weight by tibial length (Figure 1D). Compared to WT mice, FGF1 deletion slightly increased liver weight and liver weight to tibial length ratio in PBS treated controls (Figure 1C,D); however, DOX treatment markedly decreased liver weight and liver weight to tibial length ratio in both WT and FGF1-KO mice and FGF1 deletion obviously aggravated these indices’ reduction (32% vs. 47%, respectively), although no statistically significant differences were observed between WT and FGF1-KO mice (Figure 1C,D). The effects of DOX on liver damage were evaluated by measuring the plasma markers of liver injury: ALT and AST (Figure 1E,F). Compared to WT mice, FGF1 deletion marginally decreased ALT and AST in PBS treated controls (Figure 1E,F). In contrast, FGF1 deletion aggravated DOX-induced increases in both ALT and AST (11% vs. 52% and 29% vs. 63%, respectively), although no significant differences were observed between WT and FGF1-KO mice (Figure 1E,F). These findings indicate that endogenous FGF1 deletion obviously aggravates DOX-induced liver damage.

### 3.2. FGF1 Deficiency Aggravates DOX-Induced Liver Oxidative Stress

**Figure 2:**
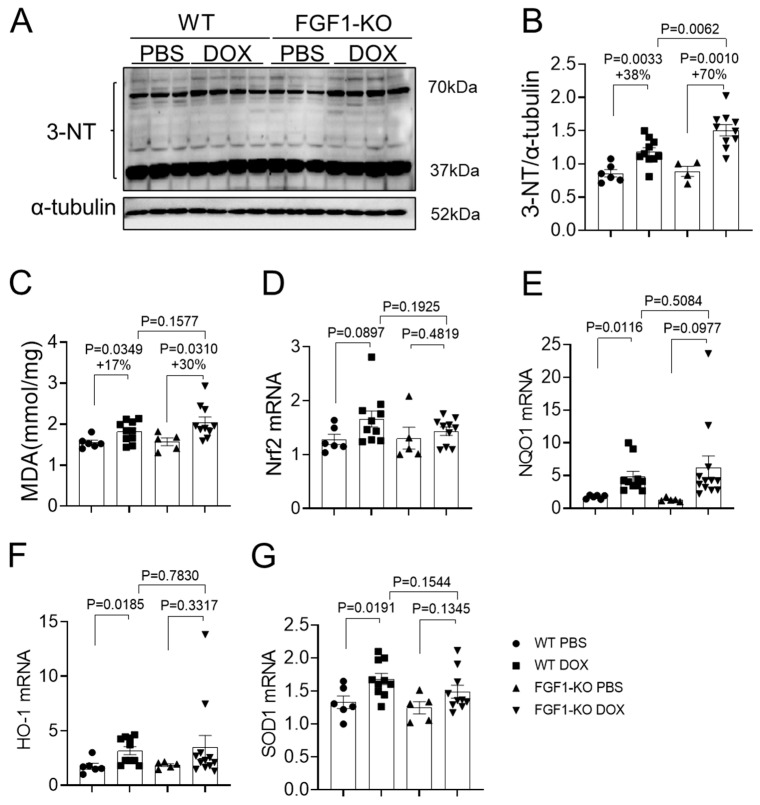
FGF1 deficiency aggravated DOX-induced liver oxidative stress. (**A**,**B**) 3-Nitrotyrosine (3-NT) expression was detected by Western blot and quantified by densitometry. (**C**) Malondialdehyde content. (**D**–**G**) Relative mRNA expression of Nrf2, NQO1, HO-1, SOD1 was detected by RT-PCR. Values represent the mean ± SEM. *p* < 0.05 was considered statistically significant. The percentage under the *p* values indicated the magnitude of increase (+) between two groups.

Oxidative stress is a primary factor for triggering the development of DOX-induced liver damage [5]. Therefore, 3-NT modification of proteins, MDA content and the expression of major antioxidant genes were determined to see the effects of endogenous FGF1 deficiency on DOX-induced oxidative stress. Western blot results showed that DOX treatment significantly increased 3-NT modification of multiple proteins in the liver of both WT and FGF1-KO mice, which were further exacerbated by endogenous FGF1 deficiency (38% vs. 70%, Figure 2A,B). Similarly, DOX treatment significantly increased hepatic MDA content in both WT mice and FGF1-KO mice, and FGF1 deficiency slightly exacerbated the elevation of MDA content (17% vs. 30%, Figure 2C). Nrf2 is one of the most important transcriptional factors in the cellular response to oxidative stress [22]. Hepatic Nrf2 expression was obviously, although not significantly (*p* = 0.0897), elevated by DOX treatment in WT mice, which could not be seen in FGF1-KO mice (Figure 2D). Consistent with this increasing trend, DOX treatment also significantly upregulated the mRNA expression of Nrf2 downstream genes including NQO1, HO-1 and SOD1 in WT mice; but these could only be seen for NQO1 expression in FGF1-KO mice, although an increasing trend could be observed for HO-1 and SOD1 expression (Figure 2D–G). These findings indicate that FGF1 deficiency aggravates DOX-induced hepatic oxidative damage, which is associated with an attenuation of Nrf2-mediated antioxidative response.

### 3.3. FGF1 Deficiency Slightly Exacerbates DOX-Induced Liver Cell Apoptosis

**Figure 3:**
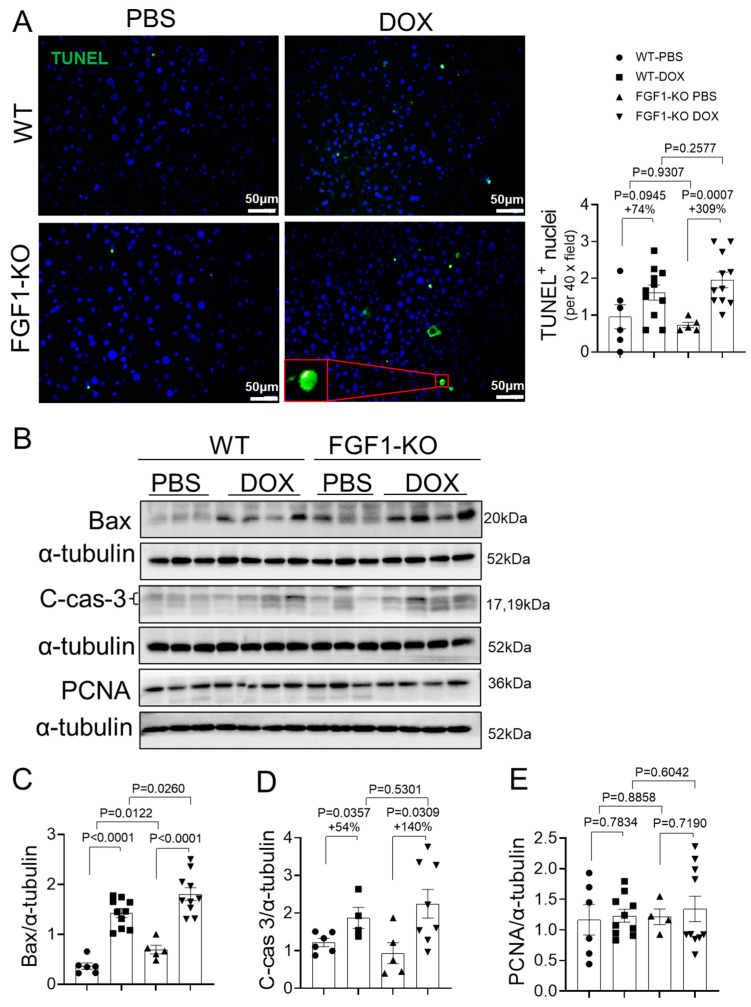
FGF1 deficiency exacerbated DOX-induced liver cell apoptosis. (**A**) Representative images of terminal deoxynucleotidyl transferase-mediated dUTP nick-end labeling (TUNEL) staining and TUNEL-positive nuclei were labeled in green, and nuclei were counterstained by DAPI. (**B**–**E**) The protein expression of BCL-2-associated X (Bax), cleaved-caspase 3 (C-cas-3) and proliferating cell nuclear antigen (PCNA) was detected by Western blot and quantified by densitometry. Values represent the mean ± SEM. *p* < 0.05 was considered statistically significant. The percentage under the *p* values indicated the magnitude of increase (+) between two groups.

Cell apoptosis is a pivotal event in the progression of DOX-induced liver damage. A TUNEL assay was used to detect apoptotic cell death in liver. After DOX treatment, TUNEL positive nuclei were significantly increased in both WT and FGF1-KO mice, which were marginally (*p* = 0.2577) exacerbated by FGF1 deletion (Figure 3A). Hepatic cell apoptosis was also evaluated by determining the expression of cell apoptotic marker BAX expression and caspase 3 cleavage. Compared to WT mice, FGF1 deletion significantly increased BAX expression in PBS treated controls, which was further exacerbated after DOX induction (Figure 3B,C). Consistently, hepatic caspase 3 cleavage was also elevated in both WT and FGF1-KO mice received DOX treatment, which was slightly exacerbated by FGF1 deletion (54% vs. 140%, Figure 3B,D). However, there were no significant differences observed between WT and FGF1-KO mice either with or without DOX treatment for a cell proliferation marker PCNA expression (Figure 3B,E). These findings indicate that endogenous FGF1 is essential for hepatic cell survival and FGF1 deficiency slightly aggravates DOX-induced liver cell apoptosis.

### 3.4. FGF1 Deficiency Does Not Aggravate DOX-Induced Liver Inflammatory Response

**Figure 4:**
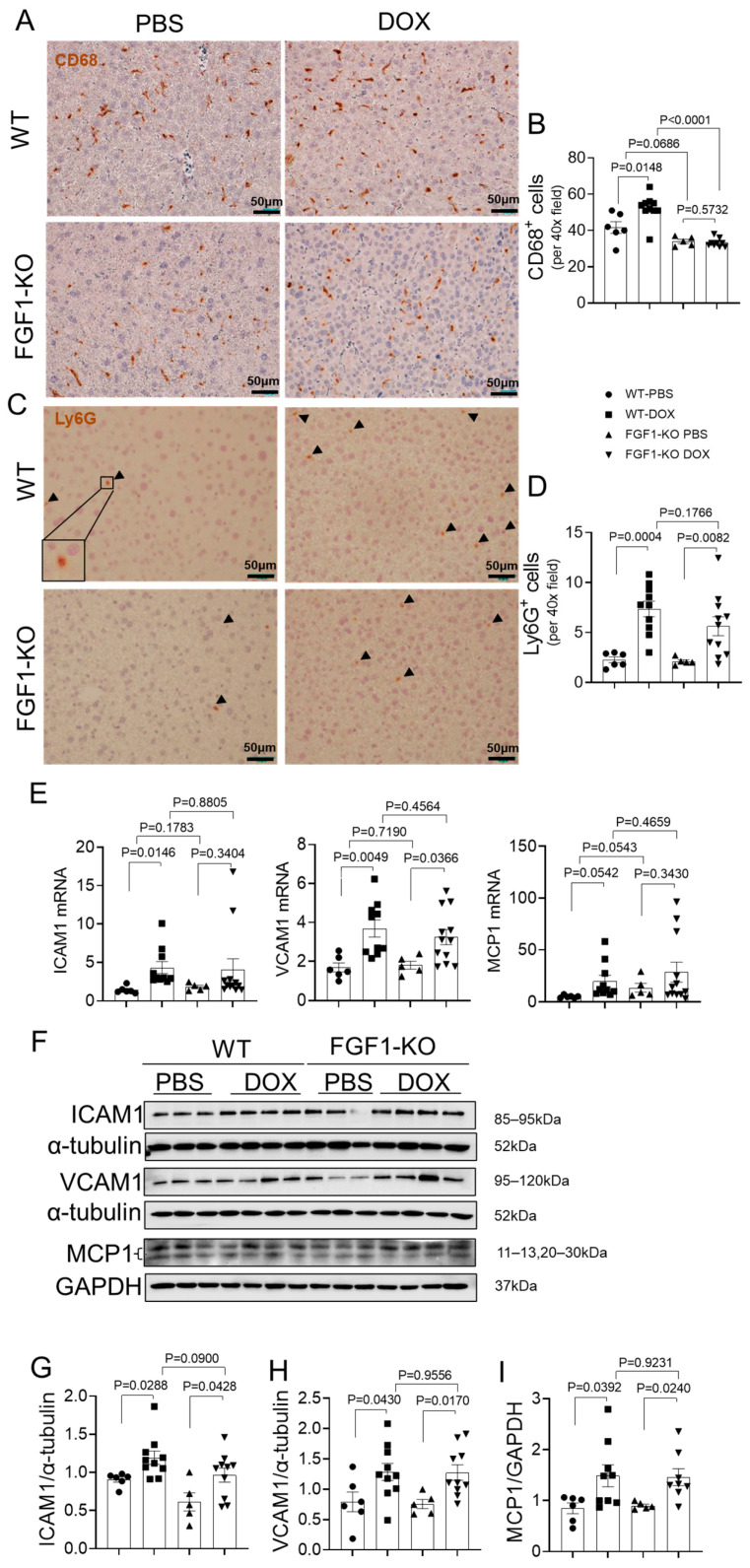
FGF1 deficiency did not aggravate DOX-induced liver inflammation. (**A**–**D**) CD68^+^ macrophages and Ly6G^+^ neutrophil cells were recognized by immunohistochemical staining and quantified as positive cells per field. (**E**) Relative mRNA expression of intercellular adhesion molecule 1 ICAM1, vascular cell adhesion molecule 1 (VCAM1) and monocyte chemoattractant protein-1 (MCP1) was detected by RT-PCR. (**F**–**I**) The protein expression of ICAM1, VCAM1 and MCP1 was detected by Western blot and quantified by densitometry. Values represent the mean ± SEM. *p* < 0.05 was considered statistically significant.

Inflammation has been considered as another critical pathological mechanism of liver damage induced by DOX treatment. To confirm whether the endogenous FGF1 deficiency aggravated DOX-induced inflammation, inflammatory cells infiltration and the expression of inflammatory cytokines in liver were investigated. CD68 [23] and Ly6G [24] are established cell surface markers of macrophages and neutrophils, respectively. Immunohistochemical staining results showed that macrophage infiltration was dramatically increased after DOX treatment in WT mice; unexpectedly, this could not be observed in FGF1-KO mice (Figure 4A,B). Ly6G staining showed that the number of neutrophil infiltration was significantly increased in both WT and FGF1-KO mice with DOX treatment, but FGF1 deletion slightly attenuated DOX-induced neutrophil infiltration compared to WT mice with DOX treatment (Figure 4C,D). The mRNA expression of pro-inflammatory cytokines ICAM1, VCAM1 and MCP1 was all upregulated after DOX treatment in both WT and FGF1-KO mice, but no significant differences could be seen between WT and FGF1-KO mice either with or without DOX treatment (Figure 4E). Similarly, the protein expression of ICAM1 (Figure 4F,G), VCAM1 (Figure 4F,H) and MCP1 (Figure 4F,I) were also upregulated in both WT and FGF1-KO mice after DOX treatment, but FGF1 deletion had no significant effects on these proteins’ expression. These results indicate that endogenous FGF1 deficiency does not aggravate DOX-induced hepatic inflammatory response.

### 3.5. FGF1 Deficiency Aggravates DOX-Induced Liver Fibrosis

**Figure 5:**
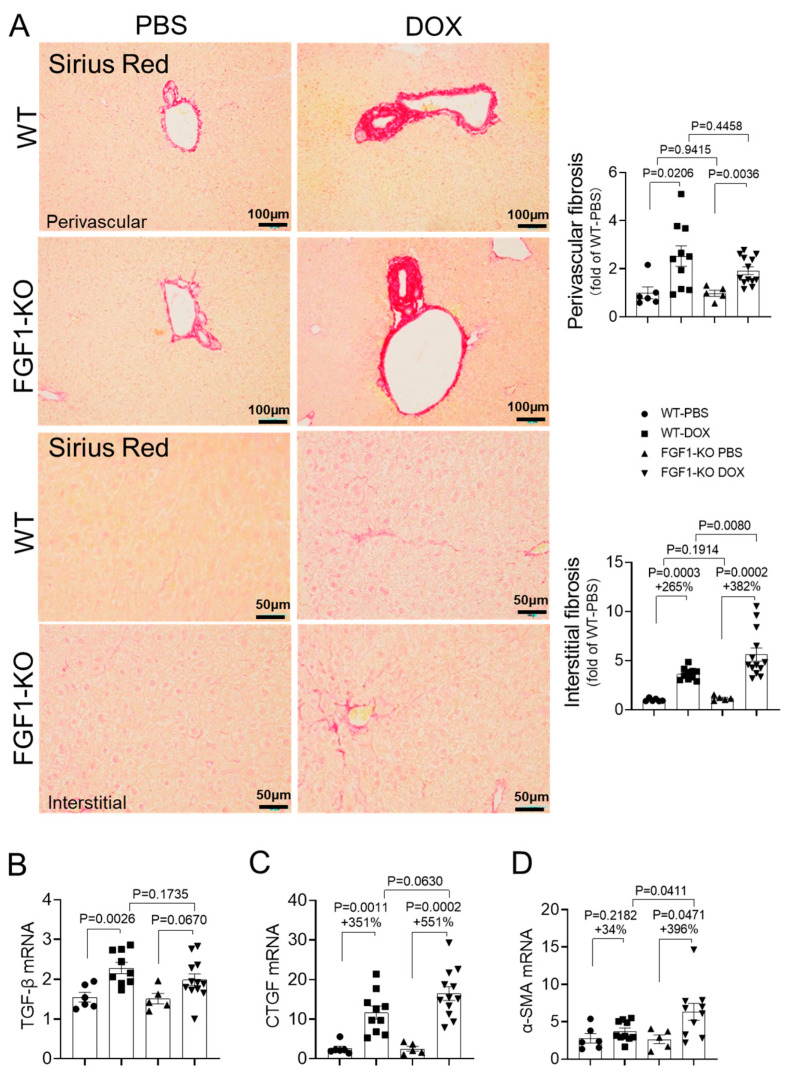
FGF1 deficiency aggravated DOX-induced liver fibrosis. (**A**) Representative images of Sirius Red staining and quantitative analysis of perivascular and interstitial fibrosis. (**B**–**D**) Relative mRNA expression of TGFβ, CTGF, and α-SMA was detected by RT-PCR. Values represent the mean ± SEM. *p* < 0.05 was considered statistically significant. The percentage under the *p* values indicated the magnitude of increase (+) between two groups.

Sirius Red staining was performed to evaluate the fibrotic changes in liver. Compared to WT mice, FGF1 deletion had no significant effects on either interstitial and perivascular fibrosis in PBS-treated controls (Figure 5A). In contrast, DOX treatment significantly induced interstitial and perivascular fibrosis in both WT and FGF1-KO mice, and FGF1 deletion markedly worsened interstitial fibrosis (265% vs. 382%), although this was not observed in perivascular fibrosis (Figure 5A). The mRNA expression of fibrotic markers was also determined by qRT-PCR. The results showed that DOX treatment obviously elevated the expression of TGF-β (Figure 5B), CTGF (Figure 5C), and α-SMA (Figure 5D) in liver tissues of both WT and FGF1-KO mice that received DOX treatment; FGF1 deletion further aggravated DOX-induced upregulation of both CTGF and α-SMA (351% vs. 551% and 34% vs. 396% in Figure 5C,D, respectively), but no significant differences were observed between WT and FGF-KO mice for TGF-β expression. These findings indicate that endogenous FGF1 deficiency aggravates DOX-induced interstitial fibrosis in liver.

## 4. Discussion

Here, for the first time, we showed that mice with FGF1 deletion were susceptible to develop DOX-induced hepatic damages in a chronic mouse experimental model, which was mirrored by the exacerbated liver weight loss, elevated plasma ALT and AST, worsened hepatic oxidative stress and cell apoptosis, and aggravated hepatic interstitial fibrosis, although the endogenous FGF1 expression in liver remained unchanged after DOX induction. Our results indicate that endogenous FGF1 plays a critical role in protecting the liver against the development of DOX-induced hepatoxicity.

Indeed, accumulating evidence indicates that FGF1 is involved in various liver diseases [21,23,25,26]. FGF1 has been shown to protect mice from acetaminophen-induced hepatoxicity through suppressing inflammation, apoptosis, oxidative stress, and endoplasmic reticulum stress [25]. FGF1 exhibited strong anti-inflammatory and antisteatotic effects in ob/ob mice that were associated with nonalcoholic fatty liver disease (NAFLD) [23]. Our recent study demonstrated that FGF1^ΔHBS^, a non-mitogenic variant of native FGF1, prevented high fat and high cholesterol (HFHC) diet-induced nonalcoholic steatohepatitis (NASH) in ApoE knockout (ApoE-KO) mice and reversed the established NAFLD in late stage db/db type 2 diabetic mice [21]. A more recent study showed that FGF1 alone or in combination with a natural product resveratrol prevented DOX-induced hepatic oxidative stress, apoptosis and inflammation [27]. Together with our results in the present study, these findings indicate that FGF1 is a potential therapeutic target for treatment of DOX-induced hepatotoxicity in cancer patients.

Liver weight loss is a primary sign of DOX-induced hepatic alteration [28]. In the present study, we observed dramatic liver weight loss induced by DOX treatment in both WT and FGF1-KO mice, which was obviously aggravated by FGF1 deletion (Figure 1), indicating that endogenous FGF1 is important for maintaining liver mass under DOX treatment conditions. The elevated ALT and AST levels in the blood are another primary indicator for DOX-induced hepatic injury [5,28]. Indeed, DOX treatment induced remarkable elevation of ALT and AST in both WT and FGF1-KO mice; although no significant differences were observed between WT and FGF1-KO mice that received DOX treatment, the elevating extents of both ALT and AST were more obvious in FGF1-KO mice than that of WT mice (Figure 1). These findings indicate that FGF1 is also an essential regulator for maintaining hepatic integrity and function under certain pathological conditions. In support of this notion, our recent study indicated that replenishment of recombinant FGF1^△HBS^ protein mitigated the elevation of plasma ALT and ALT in type 2 diabetic mice [21]; a more direct evidence from a recent study indicated that replenishment of recombinant FGF1 protein alone or in combination with resveratrol almost completely normalized plasma ALT and AST in DOX-treated mice [27].

FGF1 was reported to have antioxidative activities against diabetes associated NAFLD [21] and DOX-induced hepatoxicity [27], and hepatic oxidative stress was thought to paly causal roles in the development of DOX-induced hepatoxicity, since supplement of an antioxidant compound N-acetyl cysteine significantly attenuated DOX-induced liver damage [29,30,31]. Thus, we assumed that FGF1 deletion might exacerbate hepatic oxidative stress, contributing to the aggravated development of DOX-induced liver damage in FGF1-KO mice. As expected, in the present study both FGF1-KO and WT mice that received DOX treatment had elevated typically oxidative damage markers reflected by nitrosative modification of multiple groups of hepatic proteins and peroxidation of hepatic lipids, which were all obviously aggravated in FGF1-KO mice (Figure 2). These findings imply that endogenous FGF1 is an essential factor for tolerating DOX-induced hepatic oxidative damage. In line with our assumption, a recent study demonstrated that administration of FGF1 alone or in combination with resveratrol profoundly ameliorated DOX-induced oxidative and nitrosative damages in liver [27], indicating that replenishment exogenous FGF1 is a promising strategy for preventing DOX-induced hepatoxicity.

Oxidative stress is a pathophysiological status caused by an imbalance between production and accumulation of ROS in cells and tissues and the ability of a biological system to detoxify these reactive products [32]. Nrf2 is a critical oxidative stress responsive transcriptional factor. Nrf2 signaling pathway and subsequent induction of its downstream cytoprotective protein expression is the main cellular defense mechanism against oxidative and electrophilic stress. Therefore, interactions of Nrf2 with other signaling components regulate the efficiency of the cellular stress response [33]. Our above findings showed that hepatic oxidative stresses were amplified by FGF1 deletion after DOX induction and our recent study indicated that Nrf2 played a pivotal role in FGF1^△HBS^ protecting against NAFLD associated hepatic oxidative stresses [21]; therefore, we hypothesized that FGF1 deficiency might further impair Nrf2-mediated antioxidative signaling pathways under DOX treatment conditions. In the present study, we found that DOX treatment markedly triggered Nrf2-mediated antioxidative response pathway in livers of WT mice, as indicated by increased Nrf2 expression and up-regulation of the transcriptional expression of Nrf2 regulated downstream antioxidant genes, NQO1, HO-1 and SOD1 (Figure 2). However, this compensative response pathway was dramatically blunted by FGF1 deletion, reflected by a marginal upregulation of Nrf2 expression and its downstream gene transcription (Figure 2). Our findings demonstrated that DOX-induced oxidative stress led to liver damage, and hepatic Nrf2 and its downstream antioxidant genes were essential responsive factors to rectify DOX-induced redox imbalance, which were attenuated by FGF1 deficiency in FGF1-KO mice with DOX treatment. These findings imply that Nrf2 is an important mediator for endogenous FGF1-mediated tolerance of DOX-induced hepatic oxidative damage and replenishment of exogenous FGF1 would strengthen Nrf2-mediated compensatively antioxidative reaction against DOX-induced hepatic oxidative damage. This notion is truly evidenced by the recent study demonstrating that administration of FGF1 alone or together with resveratrol could efficiently mitigate DOX-induced hepatic oxidative and nitrosative stresses via upregulation of Nrf2-mediated antioxidative pathways [27].

Apoptosis is essential for the normal functioning and survival of most multi-cellular organisms [34]. The imbalanced redox potential triggers to a series of events resulting in cell apoptosis [34]. Accumulating evidence indicates that induction of liver cell apoptosis is a key mechanism playing critical roles in the development of DOX-induced hepatoxicity [5]. Because FGF1 deletion aggravated DOX-induced hepatic oxidative stress, we assumed that endogenous FGF1 deficiency would exacerbate DOX-induced cell apoptosis in FGF1-KO mice. Indeed, DOX treatment induced substantial apoptotic cell death as indicated by TUNEL positive nuclei staining and apoptotic signaling activation as indicated by upregulated BAX expression and caspase 3 cleavage in livers of both WT and FGF1-KO mice, which were substantially exacerbated by FGF1 deletion (Figure 3). Since DOX had no significant effects on cell proliferating maker PCNA expression in both WT and FGF1-KO mice either with or without DOX treatment (Figure 3), we have a good reason to speculate that FGF1 deficiency aggravated DOX-induced liver weight loss and injury could attribute, at least partially, to FGF1 deficiency exacerbated liver cell apoptotic cell death.

Both DOX-induced oxidative stress and cell death may trigger defensive immune responses, resulting in excessive inflammatory cell infiltration and tissue damage [5]. Thus, we assumed that FGF1 deficiency also exacerbates DOX-induced hepatic inflammatory responses. In the present study, we did observe overt inflammatory responses in both WT and FGF1-KO mice with DOX treatment as indicated by significantly increased Ly6G^+^ neutrophil infiltration and promoted expression of molecular markers that involved in inflammatory cell adhesion and migration such as ICAM1, VCAM1 and MCP1 at both mRNA and protein levels, although the elevation of CD68^+^ macrophage infiltration was only observed in WT mice (Figure 4). Unexpectedly, DOX-induced these inflammatory responses were not amplified by FGF1 deletion (Figure 4). A previous study showed that administration of recombinant FGF1 effectively improved hepatic inflammation and liver damage in both ob/ob and choline deficient diet-induced mouse models of NAFLD [23]. A recent study demonstrated that administration of FGF1 alone or in combination with resveratrol substantially mitigated DOX-induced hepatic inflammatory molecular marker expression [27]. The inconsistency of the results between our study and previous findings mentioned above imply that liver endogenous FGF1 may not directly regulate hepatic inflammatory responses and exogenous FGF1 exerts its anti-inflammatory properties through a systemic action. This assumption is partially supported by our recent study showed that administration of FGF1^ΔHBS^ improved inflammatory responses of NAFLD associated with db/db type 2 diabetes through systemic rectifying the metabolic status [21].

Chronic liver injury and/or inflammation leads to an increased collagen deposition and build-up of scar tissue, resulting in hepatic fibrosis, and controlling excessive liver injury and inflammation has great therapeutic potential for inhibiting DOX-induced progressive hepatic fibrosis [4]. We speculated that endogenous FGF1 deficiency aggravated hepatic oxidative stress and cell death would also exacerbate DOX-induced liver fibrosis. Indeed, corresponding to the aggravated hepatic oxidative stress and cell death by FGF1 deletion, DOX treatment induced more substantial collagen deposition in hepatic interstitial areas of FGF1-KO mice than that of WT mice, which was accompanied by a further upregulated expression of several essential profibrotic molecular markers such as CTGF and α-SMA (Figure 5). Unexpectedly, FGF1 deletion had no significant effects on DOX-induced perivascular collagen accumulation (Figure 5), indicating differential actions of endogenous FGF1 in regulating hepatic perivascular and interstitial extracellular matrix homeostasis. In contrast, our recent study demonstrated that administration of FGF1^ΔHBS^ mitigated both hepatic perivascular and interstitial collagen deposition in HFHC diet-induced NASH in ApoE-KO mice [21]. These contradictory findings could be explained by the differential expression patten of endogenous FGF1 protein in liver tissues: FGF1 protein was mainly detectable in the interstitial extracellular matrix area but not in endothelial cells of the hepatic artery and portal vein [35]. Therefore, endogenous FGF1 deletion only aggravated DOX-induced fibrotic responses in the interstitial tissue, while systemic administration of exogenous FGF1 protein could diffuse into both interstitial and perivascular tissues and simultaneously ameliorate DOX-induced collagen deposition in these two areas.

## 5. Conclusions

FGF1 deletion impairs Nrf2-mediated antioxidant defensive responses, resulting in exacerbated hepatic oxidative stress, which in turn aggravates hepatic cell death and injury, eventually deteriorating hepatic fibrosis and accelerating the development of DOX-induced hepatoxicity. These results may advance the application of FGF1 and FGF1△HBS variant towards clinical application for treatment of DOX-induced hepatoxicity in cancer survivors.

## Data Availability

The data presented in this study are available on request from the corresponding author.
